# Hexaaqua­aluminium(III) tris­(methane­sulfonate)

**DOI:** 10.1107/S1600536812033235

**Published:** 2012-07-28

**Authors:** Thomas Trella, Walter Frank

**Affiliations:** a Institut für Anorganische Chemie und Strukturchemie, Lehrstuhl II, Heinrich-Heine-Universität Düsseldorf, Universitätsstrasse 1, D-40225 Düsseldorf, Germany

## Abstract

The title compound, [Al(H_2_O)_6_](CH_3_SO_3_)_3_ (common name: aluminium methane­sulfonate hexa­hydrate), was crystallized from an aqueous solution prepared by the precipitation reaction of aluminium sulfate and barium methane­sulfonate. Its crystal structure is the first of the boron group methane­sulfonates to be determined. The characteristic building block is a centrosymmetric unit containing two hexa­aqua­aluminium cations that are connected to each other by two O atoms of the –SO_3_ groups in an O—H⋯O⋯H—O sequence. Further O—H⋯O hydrogen bonding links these blocks in orthogonal directions – along [010] forming a double chain array, along [10-1] forming a layered arrangement of parallel chains and along [101] forming a three-dimensional network. As indicated by the O⋯O distances of 2.600 (3)–2.715 (3) Å, the hydrogen bonds are from medium–strong to strong. A further structural feature is the arrangement of two and four methyl groups, respectively, establishing ‘hydro­phobic islands’ of different size, all positioned in a layer-like region perpendicular to [101]. The only other building block within this region is one of the –SO_3_ groups giving a local connection between the hydro­philic structural regions on both sides of the ‘hydro­phobic’ one. Thermal analysis indicates that a stepwise dehydration process starts at about 413 K and proceeds *via* the respective penta- and dihydrate until the compound completely decomposes at about 688 K.

## Related literature

For crystal structure determinations of hexa­aqua­aluminium salts, see: Andress & Carpenter (1934 ▶); Buchanan & Harris (1968 ▶); Cameron *et al.* (1990 ▶); Herpin & Sudarsanan (1965 ▶); Lazar *et al.* (1991 ▶); Lipson & Beevers (1935 ▶). For hexa­coordinated aluminium in compounds with chelating ligands, see: Hon & Pfluger (1973 ▶); McClelland (1975 ▶); Taylor (1978 ▶). For ligand properties of methane­sulfonate, see: Paul *et al.* (1974 ▶). For physical and chemical properties of methane­sulfonates in general, see: Aricó *et al.* (2001 ▶); Gernon *et al.* (1999 ▶); Trella *et al.* (2012 ▶); Wang, Song, Jiang & Gong (2009 ▶). For other metal(III) methane­sulfonates, see: Aricó *et al.* (1997 ▶); Aricó *et al.* (2001 ▶); Frank & Wallus (2006 ▶); Lindqvist-Reis *et al.* (2006 ▶); Wickleder (2001 ▶); Wickleder & Müller (2004 ▶). For spectroscopic data of other methane­sulfonates, see: Capwell *et al.* (1968 ▶); Reiss & Meyer (2011 ▶); Stahlberg *et al.* (1967 ▶). For methane­sulfonates in catalysis, see: Wang, Jiang, Gong & Wang (2003 ▶); Wang, Jiang, Gong, Wang & Liu (2003 ▶); Wang, Tian, Song & Jiang (2009 ▶); Zhang (2007 ▶). For graph-set analysis, see Etter *et al.* (1990 ▶).
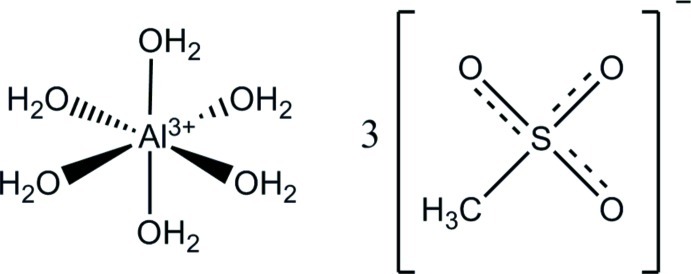


## Experimental

### 

#### Crystal data

[Al(H_2_O)_6_](CH_3_O_3_S)_3_
*M* *_r_* = 420.39
Monoclinic, 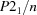

*a* = 16.4677 (9) Å
*b* = 6.4239 (4) Å
*c* = 17.4295 (8) Å
β = 117.035 (5)°
*V* = 1642.34 (17) Å^3^
*Z* = 4
Mo *K*α radiation
μ = 0.58 mm^−1^
*T* = 173 K
0.38 × 0.12 × 0.03 mm

#### Data collection

Stoe IPDS diffractometer
Absorption correction: multi-scan (*SHELXTL*; Sheldrick, 2008 ▶) *T*_min_ = 0.811, *T*_max_ = 0.983
20432 measured reflections
2874 independent reflections
2076 reflections with *I* > 2σ(*I*)
*R*_int_ = 0.057

#### Refinement

*R*[*F* ^2^ > 2σ(*F* ^2^)] = 0.031
*wR*(*F* ^2^) = 0.073
*S* = 1.12
2874 reflections
262 parameters
2 restraints
H atoms treated by a mixture of independent and constrained refinement
Δρ_max_ = 0.43 e Å^−3^
Δρ_min_ = −0.29 e Å^−3^

### 

Data collection: *IPDS Software* (Stoe & Cie, 2000 ▶); cell refinement: *IPDS Software*; data reduction: *IPDS Software*; program(s) used to solve structure: *SHELXS97* (Sheldrick, 2008 ▶); program(s) used to refine structure: *SHELXL97* (Sheldrick, 2008 ▶); molecular graphics: *DIAMOND* (Brandenburg, 2010 ▶) and *SHELXTL* (Sheldrick, 2008 ▶); software used to prepare material for publication: *SHELXL97*.

## Supplementary Material

Crystal structure: contains datablock(s) I, global. DOI: 10.1107/S1600536812033235/gg2089sup1.cif

Structure factors: contains datablock(s) I. DOI: 10.1107/S1600536812033235/gg2089Isup2.hkl

Additional supplementary materials:  crystallographic information; 3D view; checkCIF report

## Figures and Tables

**Table 1:** Hydrogen-bond geometry (Å, °)

*D*—H⋯*A*	*D*—H	H⋯*A*	*D*⋯*A*	*D*—H⋯*A*
O10—H1⋯O8^i^	0.85 (4)	1.81 (4)	2.659 (3)	174 (3)
O10—H2⋯O7	0.83 (4)	1.80 (4)	2.627 (3)	176 (4)
O11—H3⋯O1	0.84 (3)	1.78 (3)	2.615 (3)	169 (3)
O11—H4⋯O5^ii^	0.81 (3)	1.87 (3)	2.683 (3)	175 (3)
O12—H5⋯O1^iii^	0.77 (2)	1.84 (2)	2.608 (2)	171 (3)
O12—H6⋯O2	0.85 (3)	1.87 (3)	2.713 (2)	174 (3)
O13—H7⋯O3^iv^	0.79 (2)	1.87 (2)	2.648 (2)	169 (4)
O13—H8⋯O4^v^	0.89 (4)	1.82 (4)	2.715 (3)	177 (3)
O14—H9⋯O9^iii^	0.79 (4)	1.82 (4)	2.600 (3)	174 (4)
O14—H10⋯O4	0.88 (4)	1.84 (4)	2.714 (3)	172 (3)
O15—H11⋯O6^vi^	0.78 (4)	1.89 (4)	2.667 (3)	171 (4)
O15—H12⋯O5	0.87 (4)	1.81 (4)	2.674 (3)	169 (3)

Symmetry codes: (i) 
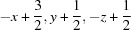
; (ii) 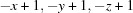; (iii) 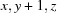; (iv) 
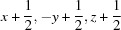
; (v) 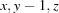; (vi) 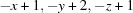.

**Fig. 1.**
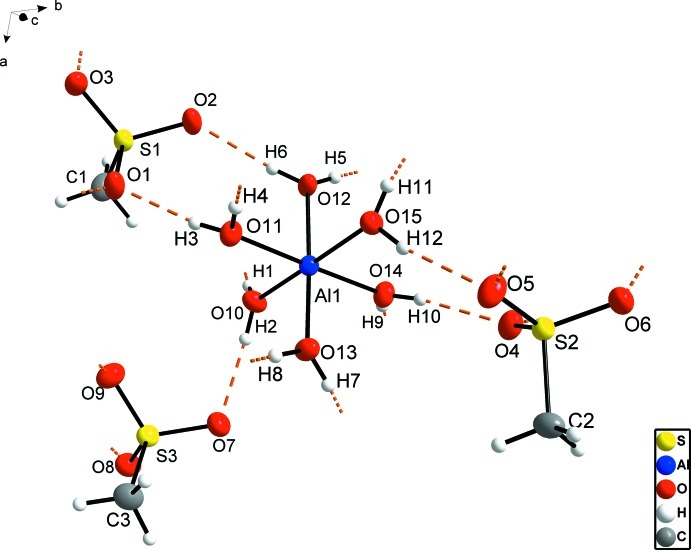
: Diagram of the chosen asymmetric unit of the crystal structure of I showing the atom numbering scheme; hydrogen atoms are drawn with an arbitrary radius and displacement ellipsoids are drawn at the 50% probability level. H atom labels have been omitted for methyl groups. Dashed lines indicate hydrogen bonds.

**Fig. 2.**
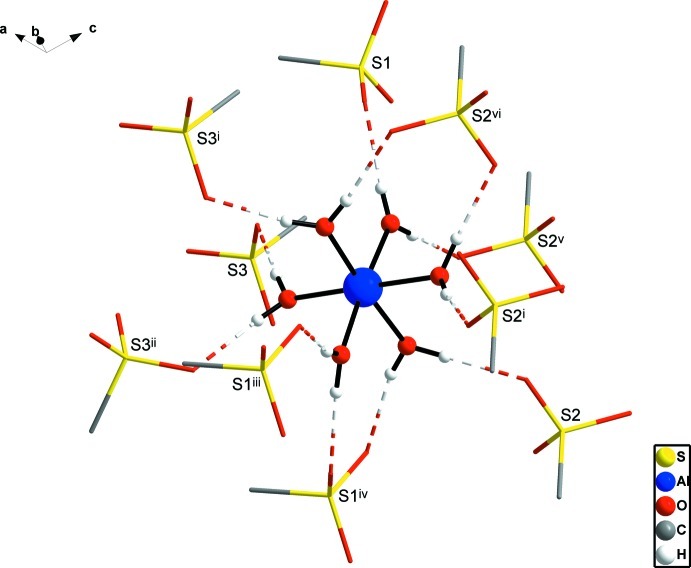
: The hexaaquaaluminium cation with its highly asymmetric coordination environment of ten sulfonate anions. [Symmetry codes: (i) x, 1 + y, z; (ii) 1.5–x, 0.5 + y, 1.5–z; (iii) -0.5 + y, 1.5–y, -0.5 + z; (iv) -0.5 + x, 0.5–y, -0.5 + z; (v) 1–x, –y, 2–z; (vi) 1–x, 1–y, 2–z].

**Fig. 3.**
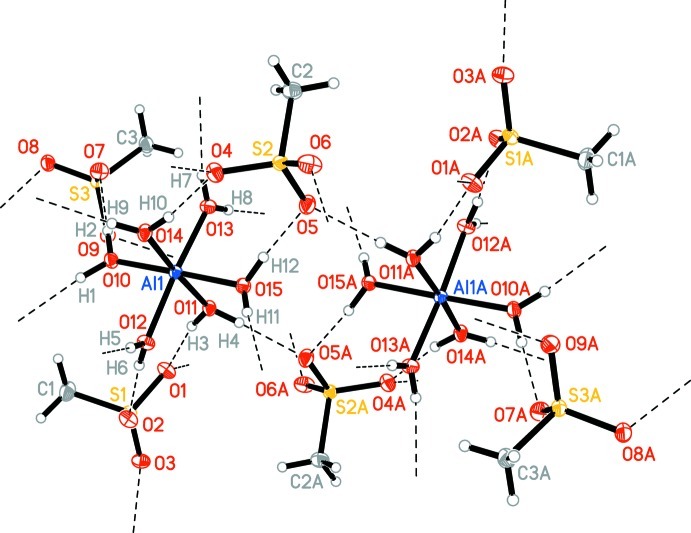
: The isolated defining segment of the double chain structural motif in the solid of I viewed along the chain propagation direction [0 1 0]; the complete system of hydrogen bonding is indicated by dashed lines. [Symmetry code: (A) 1–x, 1–y, 1–z].

**Fig. 4.**
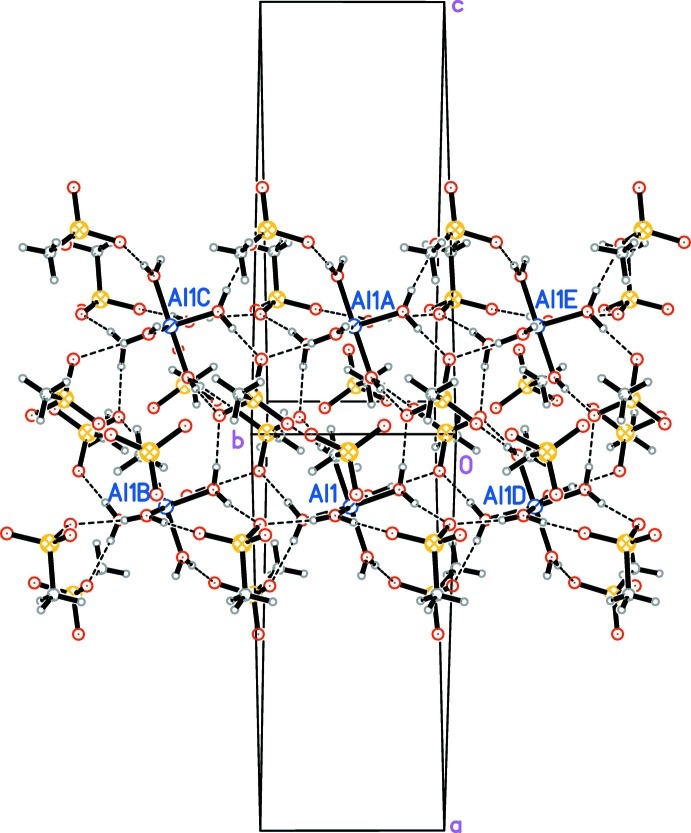
: The hexaaquaaluminium cations with the central atoms Al1 and Al1A define a double chain structure with the backbone along [0 1 0]. Connecting elements in this direction are the methanesulfonate anions with atoms S2 and S2A and their translation symmetry equivalents. The viewing direction (close to [1 0 1]) is approximately perpendicular to the chain propagation direction. [Symmetry codes: (A) 1–x, 1–y, 1–z; (B) x, y + 1, z; (C) 1–x, –y, 1–z; (d) x, y–1, z; (E) 1–x, –y, 1–z].

**Fig. 5.**
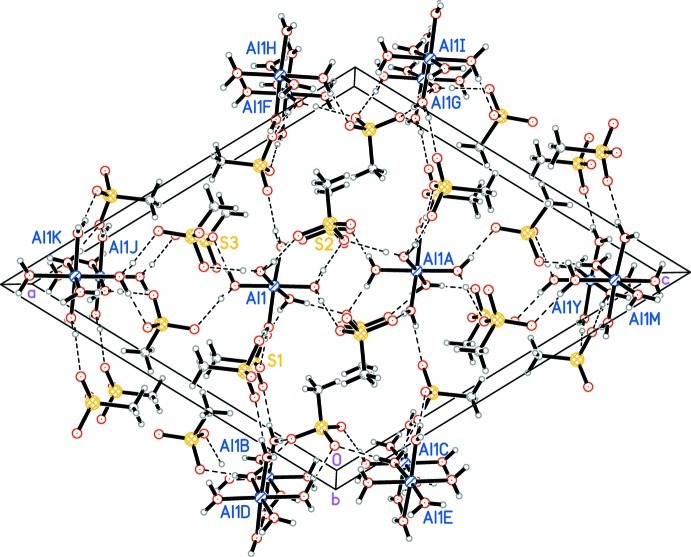
: Packing diagram of I; viewing direction [0 - 1 0]. Connection to neighbouring double chains is given by the anions with S1 and S1A along [1 0 1] and by the anions with S3 and S3A along [1 0 - 1]. [Symmetry codes: (A) 1–x, 1–y, 1–z; (B) 0.5–x, -0.5 + y, 0.5–z; (C) 0.5–x, 0.5 + y, 0.5–z; (D) 1.5–x, -0.5 + y, 0.5–z; (E) 1.5–x, -0.5 + y, 1.5–z; (F) 1.5–x, 0.5 + y, 0.5–z; (G) 1.5–x, 0.5 + y, 1.5–z; (H) -0.5 + x, 0.5–y, -0.5 + z; (I) -0.5 + x, 0.5–y, 0.5 + z; (J) -0.5 + x, 1.5–y, -0.5 + z; (K) -0.5 + x, 1.5–y, 0.5 + z; (Y) 0.5 + x, 0.5–y, 0.5 + z; (M) 0.5 + x, 1.5–y, 0.5 + z].

## supplementary crystallographic information

### Comment

Regarding catalysis and crystal engineering, the properties of methanesulfonates as eco-friendly Lewis acids (Wang *et al.*, 2009) and as components of layered inorganic-organic hybrid materials (Frank & Wallus, 2006), respectively, have been increasingly investigated during the last decade. Methanesulfonates are throughout excellently water-soluble, stable up to about 400 °C and practically inert against hydrolysing, oxidizing or reductive agents (Gernon *et al.*, 1999; Aricó *et al.*, 2001).

According to some few initial studies that show aluminium methanesulfonate to have a high catalytic activity for certain syntheses of esters and acetals, the compound is supposed to be an attractive halogen free substitute for the frequently used aluminium trifluoromethanesulfonate, for instance (Wang *et al.*, 2003, 2003; Zhang, 2007).

While in the case of scandium (Wickleder & Müller, 2004; Lindqvist-Reis *et al.*, 2006) and several lanthanides (Aricó *et al.*, 1997, 2001; Wickleder, 2001) the structures of the methanesulfonates crystallized from aqueous solutions at room temperature have already been determined, structural information for the salts of the triels still has to be collected. In general, chemistry and structures of hexaaquatriel compounds with mineral acid anions and in particular of those of the group's most important element aluminium are quite well known – most representative examples are the halides or the alums (Andress & Carpenter, 1934; Buchanan & Harris, 1968; Lipson & Beevers, 1935).

Because water is a better coordinating ligand as compared to methanesulfonate (Paul *et al.*, 1974), aluminium methanesulfonate not unexpectedly crystallized as the hexaaqua complex compound I from the aqueous solution we prepared by the precipitation reaction of aluminium sulfate and barium methanesulfonate. In contrast, recent work of other groups stated the tetrahydrate to be the only product crystallizing from aqueous solution (Wang *et al.*, 2003, 2003, 2009; Zhang, 2007). With chelating anions like oxalate (Taylor, 1978) and acetylacetonate (Hon & Pfluger, 1973; McClelland, 1975) no aqua but tris(chelate) complexes with these counterions directly engaged in the sixfold coordination about the aluminium cation are formed.

Fig. 1 shows the chosen asymmetric unit of the crystal structure of I, which contains one hexaaquaaluminium cation and three methanesulfonate ions, all in general position. The Al—O distances are in the range 1.851 (2)–1.895 (2) Å. The bond lengths and angles of the sulfonate ions are as expected with S—O distances of 1.4413 (18)–1.4737 (18) Å and S—C distances of 1.741 (3)–1.762 (3) Å. On the one hand, all three oxygen atoms of each anion are involved in at least one O—H···O hydrogen bond to an aqua ligand, on the other hand, each aqua ligand is engaged in two such bonds. In total, the hexaaquaaluminium cation is surrounded by ten methanesulfonate ions (Fig. 2). O···O distances of 2.600 (3)–2.715 (3) Å indicate medium strong to strong hydrogen bonding. The significant strength of these interactions is further reflected by the dispersion of O—Al—O angles (86.71 (8)–92.26 (9)°) as well as by the strong directing influence with respect to the orientation of the aqua ligands' hydrogen atoms. The geometric parameters of the hexaaquaaluminium cation are in agreement with the results of the X-ray and neutron diffraction analyses done on hexaaquaaluminium chloride (Buchanan & Harris, 1968) as well as with the structures of the respective hexaaquaaluminium hydroxymethanesulfonate (Cameron *et al.*, 1990) and the nitrate (Herpin & Sudarsanan, 1965; Lazar *et al.*, 1991).

Despite the intrinsic threefold symmetry of all the ionic components as well as the quasi-hexagonal metric of the unit cell, there is no obvious relation of the solid state structure of I to any kind of close packing of ionic components. As illustrated in Figs. 3–5, the solid can easily be described as a three-dimensional network 'constructed' by electrostatic assisted O—H···O hydrogen bonding in three orthogonal directions. The bonds are parts of arrangements of the types —O—H···O···H—O— or —O—H···O—S—O···H—O—. In terms of graph-set analysis (Etter *et al.*, 1990), there are entirely twelve motifs to be considered for a complete description of the hydrogen bond pattern. As no bifurcated bonds are present, these motifs can easily be denominated using the labels of the H atoms involved. The results of the graph set analysis presented here are mainly restricted to first order aspects. Main paths of O—H···O hydrogen bonding along the orthogonal directions [0 1 0], [1 0 - 1] and [1 0 1] can be characterized by the graph sets C^1^_2_(6) (H8, H10), C^3^_4_(14) (H1, H9, H4, H12) and C^2^_2_(8) (H6, H7), respectively. Some parts of these paths coincide with components of cyclic graphs (*R*^2^_4_(12) (H4, H12), *R*^4^_4_(14) (H1, H9)).

From the 'molecular' point of view, the characteristic building block of the solid is a centrosymmetric unit composed of two hexaaquaaluminium cations that are connected by single O atoms of two —SO_3_ groups (*R*^2^_4_(12) (H4, H12)) (Fig. 3). By translation along [0 1 0] a double chain structure results. As mentioned above, the main path of hydrogen bonding in this direction is characterized by the graph set C^1^_2_(6) with one acceptor O atom being part of the —SO_3_ group including S2 (Fig. 4). Connecting elements perpendicular to the chain propagation direction are the methanesulfonate anions with atom S3 ([1 0 - 1]) and atom S1 ([1 0 1]) (Fig. 5).

Usually, methanesulfonates tend to build layer-like structures with strictly separated hydrophilic and hydrophobic areas, the latter consisting of methyl groups connected by van der Waals forces (Trella *et al.*, 2012). Although the structure of I is best described as a three-dimensional network, there is an obvious relationship to this principle of construction: Two and four methyl groups, respectively, establish 'hydrophobic islands' of different size, all positioned in a layer-like region perpendicular to [1 0 1] (Fig. 5). The only other building block within this region is the —SO_3_ group including S1, giving local connection between the hydrophilic structural regions at both sides of the 'hydrophobic' one.

### Experimental

Hexaaquaaluminium methanesulfonate, [Al(H_2_O)_6_][CH_3_SO_3_]_3_, was prepared by the precipitation reaction of 1.00 g (1.50 mmol) aluminium sulfate octakaidecahydrate with 1.60 g (4.50 mmol) barium methanesulfonate sesquihydrate in 10 ml distilled water at room temperature. After removing barium sulfate, thin, hygroscopic, platelet-like crystals of I were grown from the colourless solution by slow evaporation of the solvent at room temperature.

A CHS analysis was performed with a Euro EA elemental analyser (HEKAtech GmbH). Calculated: C: 8.57%, H: 5.04%, S: 22.88%. Found: C: 7.34%, H: 5.05%, S: 21.12%.

Thermogravimetric and differential thermal analyses were done with a Netzsch STA 449 C Jupiter with a weight sensitivity of < 0.1 µg. The measurement was carried out in a nitrogen atmosphere (purge rate: 80 ml/min) at a heating rate of 5 C°/min from 20 °C to 660 °C. The dehydration process involves three steps. The loss of one water molecule happens not before 143 °C, pointing out the stability of the hexaaqua complex. Due to the removal of three further water molecules until 212 °C, Al(CH_3_SO_3_)_3_. 2H_2_O is formed. At 306 °C the complete dehydration has taken place. Comparable to all other known metal methanesulfonates showing final decomposition in the range of 400 to 500 °C (Aricó *et al.*, 2001; *M*. Wang *et al.*, 2009), aluminium methanesulfonate decomposes when temperature reaches 415 °C.

A Raman spectrum was recorded using a Bruker MULTIRAM spectrometer; Nd:YAG-Laser at 1064 nm; RT-InGaAS-detector; 4000–70 cm^-1^: 3026(ν(C—H), m), 2944(ν(C—H), s), 1426(δ(C—H), m), 1203(w), 1165(w), 1132(w), 1053(ν(S—O), *versus*), 996(w), 969(w), 790(ν(C—S), s), 550(δ(S—O), m), 357(*m*), 342(ρ(S—O), m), 150(w), 99(w).

IR data were collected on a Biorad/Digilab Excalibur FTS 3500 spectrometer using a MIRacle^TM^ single reflection HATR unit; 4000–560 cm^-1^: 3021(ν(C—H), s), 2944(ν(C—H), s), 2506(*m*), 1681(δ(O—H), m), 1652(δ(O—H), m), 1420(δ_as_(C—H), w), 1341(δ_s_(C—H), w), 1190(ν_as_(S—O), s), 1158(ν_as_(S—O), s), 1132(ν_as_(S—O), s), 1040(ν_s_(S—O), s), 990(*m*), 966(*m*), 928(*m*),779(ν(C—S), m), 704(*m*), 635(*m*). Within the spectrum, the stretch vibrations of the C—H bond have partially interfered with a broad, undefined band caused by O–H stretch vibrations between 3100 and 3500 cm^-1^. Band assignments were made by comparison with results of earlier work done on diisopropylammonium (Reiss & Meyer, 2011), lead (Stahlberg *et al.*, 1967), alkaline metal (Capwell *et al.*, 1968) and lanthanide methanesulfonate(*s*) (Aricó *et al.*, 2001).

### Refinement

A single-crystal suitable for structure determination was harvested from the mother liquor, directly transferred into the cooling stream of a Stoe *IPDS* diffractometer and investigated at -100 (2) °C. Thirteen reflections were excluded from the experiment, with one effected by the beam stop and twelve from the Lorentz zone of the one circle diffraction experiment, including two strong ones.

All H atom positions were located in difference Fourier maps. Positional parameters of hydrogen atoms belonging to water molecules were refined. In case of H5 and H7 the distance to the respective parent atoms O12 and O13 was restrained to 0.83 Å with a standard uncertainty of 0.03 Å. H atoms of the —CH_3_ groups were treated applying angle constraints (H—C—H 109.5°; S—C—H 109.5°). They were free to rotate about the S—C bond and additionally the C—H distances were allowed to vary, with the same shifts being applied along all the C—H bonds of a group. Anisotropic displacement parameters of all non-hydrogen atoms and individual isotropic displacement parameters for all H atoms were refined.

### Crystal data

**Table:**

[Al(H_2_O)_6_](CH_3_O_3_S)_3_	*F*(000) = 880
*M**_r_* = 420.39	-
Monoclinic, *P*2_1_/*n*	*D*_x_ = 1.700 Mg m^−^^3^
Hall symbol: -P 2yn	Mo *K*α radiation, λ = 0.71073 Å
*a* = 16.4677 (9) Å	Cell parameters from 8000 reflections
*b* = 6.4239 (4) Å	θ = 4.7–23.2°
*c* = 17.4295 (8) Å	µ = 0.58 mm^−^^1^
β = 117.035 (5)°	*T* = 173 K
*V* = 1642.34 (17) Å^3^	Plate, colourless
*Z* = 4	0.38 × 0.12 × 0.03 mm

### Data collection

**Table:**

Stoe IPDS diffractometer	2874 independent reflections
Radiation source: fine-focus sealed tube	2076 reflections with *I* > 2σ(*I*)
Graphite monochromator	*R*_int_ = 0.057
Detector resolution: 6.67 pixels mm^-1^	θ_max_ = 25.0°, θ_min_ = 2.3°
φ–scans	*h* = −19→19
Absorption correction: multi-scan (*SHELXTL*; Sheldrick, 2008)	*k* = −7→7
*T*_min_ = 0.811, *T*_max_ = 0.983	*l* = −20→20
20432 measured reflections	

### Refinement

**Table:**

Refinement on *F*^2^	Primary atom site location: structure-invariant direct methods
Least-squares matrix: full	Secondary atom site location: difference Fourier map
*R*[*F*^2^ > 2σ(*F*^2^)] = 0.031	Hydrogen site location: difference Fourier map
*wR*(*F*^2^) = 0.073	H atoms treated by a mixture of independent and constrained refinement
*S* = 1.12	*w* = 1/[σ^2^(*F*_o_^2^) + (0.035*P*)^2^] where *P* = (*F*_o_^2^ + 2*F*_c_^2^)/3
2874 reflections	(Δ/σ)_max_ = 0.001
262 parameters	Δρ_max_ = 0.43 e Å^−^^3^
2 restraints	Δρ_min_ = −0.29 e Å^−^^3^

### Special details

**Table:**

Geometry. All e.s.d.'s (except the e.s.d. in the dihedral angle between two l.s. planes) are estimated using the full covariance matrix. The cell e.s.d.'s are taken into account individually in the estimation of e.s.d.'s in distances, angles and torsion angles; correlations between e.s.d.'s in cell parameters are only used when they are defined by crystal symmetry. An approximate (isotropic) treatment of cell e.s.d.'s is used for estimating e.s.d.'s involving l.s. planes.
Refinement. Refinement of *F*^2^ against ALL reflections. The weighted *R*-factor *wR* and goodness of fit *S* are based on *F*^2^, conventional *R*-factors *R* are based on *F*, with *F* set to zero for negative *F*^2^. The threshold expression of *F*^2^ > σ(*F*^2^) is used only for calculating *R*-factors(gt) *etc*. and is not relevant to the choice of reflections for refinement. *R*-factors based on *F*^2^ are statistically about twice as large as those based on *F*, and *R*- factors based on ALL data will be even larger.

### Fractional atomic coordinates and isotropic or equivalent isotropic displacement parameters (Å^2^)

**Table:**

	*x*	*y*	*z*	*U*_iso_*/*U*_eq_	
S1	0.42486 (4)	0.07708 (9)	0.14732 (4)	0.02005 (16)	
S2	0.65617 (4)	0.97454 (9)	0.60472 (4)	0.02049 (16)	
S3	0.82913 (4)	0.04636 (9)	0.38082 (4)	0.02131 (16)	
Al1	0.59239 (4)	0.50961 (11)	0.37580 (5)	0.01734 (17)	
O1	0.47670 (12)	−0.0109 (3)	0.23435 (11)	0.0270 (4)	
O2	0.40427 (12)	0.2956 (2)	0.15149 (11)	0.0267 (4)	
O3	0.34515 (11)	−0.0492 (3)	0.09627 (12)	0.0278 (4)	
O4	0.67367 (12)	1.0148 (3)	0.53116 (11)	0.0274 (4)	
O5	0.59731 (12)	0.7901 (3)	0.58804 (12)	0.0309 (5)	
O6	0.62196 (12)	1.1551 (3)	0.62981 (13)	0.0349 (5)	
O7	0.82038 (12)	0.2633 (3)	0.40091 (12)	0.0297 (4)	
O8	0.89366 (12)	0.0213 (3)	0.34588 (11)	0.0278 (4)	
O9	0.74081 (12)	−0.0488 (3)	0.32608 (12)	0.0300 (4)	
O10	0.65563 (13)	0.4196 (3)	0.31723 (13)	0.0235 (4)	
H1	0.644 (2)	0.450 (5)	0.266 (2)	0.036 (9)*	
H2	0.709 (3)	0.375 (5)	0.345 (2)	0.054 (11)*	
O11	0.53554 (13)	0.2484 (3)	0.36416 (13)	0.0214 (4)	
H3	0.5226 (19)	0.168 (5)	0.322 (2)	0.029 (8)*	
H4	0.4966 (19)	0.230 (4)	0.3798 (18)	0.021 (8)*	
O12	0.49533 (12)	0.5926 (3)	0.27037 (12)	0.0217 (4)	
H5	0.489 (2)	0.707 (4)	0.254 (2)	0.037 (9)*	
H6	0.471 (2)	0.496 (5)	0.234 (2)	0.035 (9)*	
O13	0.68364 (12)	0.4112 (3)	0.48197 (12)	0.0231 (4)	
H7	0.7314 (18)	0.462 (5)	0.511 (2)	0.048 (10)*	
H8	0.678 (2)	0.282 (6)	0.498 (2)	0.056 (11)*	
O14	0.64698 (13)	0.7730 (3)	0.39399 (14)	0.0236 (4)	
H9	0.675 (2)	0.819 (6)	0.371 (3)	0.055 (12)*	
H10	0.650 (2)	0.852 (5)	0.436 (2)	0.044 (10)*	
O15	0.52616 (14)	0.6042 (3)	0.43396 (13)	0.0218 (4)	
H11	0.481 (2)	0.666 (5)	0.411 (2)	0.046 (11)*	
H12	0.554 (2)	0.651 (5)	0.487 (3)	0.053 (11)*	
C1	0.4976 (2)	0.0604 (5)	0.0999 (2)	0.0355 (7)	
H1A	0.4669 (7)	0.111 (3)	0.0418 (10)	0.051 (10)*	
H1B	0.5151 (10)	−0.082 (2)	0.0997 (10)	0.034 (8)*	
H1C	0.5510 (11)	0.143 (3)	0.1321 (8)	0.037 (8)*	
C2	0.76073 (18)	0.9109 (4)	0.69268 (18)	0.0298 (6)	
H2A	0.8019 (8)	1.022 (2)	0.7039 (8)	0.029 (7)*	
H2B	0.7846 (7)	0.790 (3)	0.6798 (5)	0.039 (8)*	
H2C	0.7517 (3)	0.886 (3)	0.7415 (9)	0.039 (9)*	
C3	0.87409 (18)	−0.0882 (4)	0.47970 (17)	0.0296 (6)	
H3A	0.9326 (10)	−0.0398 (19)	0.5152 (7)	0.032 (8)*	
H3B	0.8764 (10)	−0.230 (2)	0.4698 (2)	0.037 (8)*	
H3C	0.8370 (8)	−0.066 (2)	0.5063 (7)	0.022 (7)*	

### Atomic displacement parameters (Å^2^)

**Table:**

	*U*^11^	*U*^22^	*U*^33^	*U*^12^	*U*^13^	*U*^23^
S1	0.0225 (3)	0.0177 (3)	0.0182 (3)	0.0007 (2)	0.0077 (3)	−0.0005 (2)
S2	0.0203 (3)	0.0211 (3)	0.0199 (3)	0.0000 (2)	0.0089 (3)	−0.0015 (2)
S3	0.0220 (3)	0.0238 (3)	0.0200 (3)	0.0004 (2)	0.0111 (3)	0.0012 (2)
Al1	0.0179 (4)	0.0166 (4)	0.0179 (4)	−0.0005 (3)	0.0084 (3)	−0.0002 (3)
O1	0.0356 (10)	0.0181 (9)	0.0206 (9)	0.0014 (7)	0.0068 (8)	−0.0008 (7)
O2	0.0331 (10)	0.0183 (9)	0.0243 (10)	0.0036 (7)	0.0093 (8)	−0.0012 (7)
O3	0.0254 (9)	0.0235 (9)	0.0273 (10)	−0.0007 (7)	0.0058 (8)	−0.0025 (8)
O4	0.0362 (10)	0.0225 (9)	0.0250 (10)	−0.0050 (8)	0.0150 (8)	−0.0011 (8)
O5	0.0334 (10)	0.0349 (11)	0.0307 (11)	−0.0126 (8)	0.0200 (9)	−0.0100 (8)
O6	0.0296 (10)	0.0349 (11)	0.0329 (11)	0.0088 (8)	0.0079 (9)	−0.0100 (9)
O7	0.0287 (10)	0.0273 (10)	0.0322 (11)	0.0042 (8)	0.0132 (9)	−0.0020 (8)
O8	0.0279 (9)	0.0335 (10)	0.0247 (10)	0.0028 (8)	0.0145 (8)	0.0025 (8)
O9	0.0275 (9)	0.0356 (11)	0.0261 (10)	−0.0073 (8)	0.0114 (8)	0.0002 (8)
O10	0.0244 (10)	0.0282 (10)	0.0203 (11)	0.0064 (8)	0.0123 (9)	0.0036 (8)
O11	0.0248 (10)	0.0212 (9)	0.0211 (10)	−0.0032 (7)	0.0129 (9)	−0.0028 (8)
O12	0.0245 (9)	0.0148 (9)	0.0203 (10)	0.0005 (8)	0.0054 (8)	0.0009 (8)
O13	0.0216 (10)	0.0204 (10)	0.0225 (10)	−0.0023 (8)	0.0058 (8)	0.0028 (8)
O14	0.0287 (10)	0.0213 (9)	0.0248 (11)	−0.0058 (7)	0.0157 (9)	−0.0025 (8)
O15	0.0215 (9)	0.0245 (9)	0.0205 (10)	0.0019 (8)	0.0106 (8)	−0.0026 (8)
C1	0.0380 (16)	0.0380 (17)	0.0393 (18)	−0.0076 (13)	0.0253 (14)	−0.0083 (13)
C2	0.0254 (13)	0.0299 (14)	0.0294 (16)	0.0038 (12)	0.0084 (12)	0.0033 (12)
C3	0.0312 (15)	0.0337 (16)	0.0256 (15)	0.0034 (12)	0.0143 (13)	0.0060 (12)

### Geometric parameters (Å, º)

**Table:**

S1—O1	1.4737 (18)	O11—H3	0.84 (3)
S1—O2	1.4535 (17)	O11—H4	0.81 (3)
S1—O3	1.4530 (18)	O12—H5	0.77 (2)
S1—C1	1.741 (3)	O12—H6	0.85 (3)
S2—O4	1.459 (2)	O13—H7	0.79 (2)
S2—O5	1.4737 (18)	O13—H8	0.89 (4)
S2—O6	1.4413 (19)	O14—H9	0.79 (4)
S2—C2	1.756 (3)	O14—H10	0.88 (4)
S3—O7	1.4599 (18)	O15—H11	0.78 (4)
S3—O8	1.4539 (19)	O15—H12	0.87 (4)
S3—O9	1.4623 (18)	C1—H1A	0.9599
S3—C3	1.762 (3)	C1—H1B	0.9599
Al1—O10	1.851 (2)	C1—H1C	0.9599
Al1—O11	1.8868 (19)	C2—H2A	0.9411
Al1—O12	1.8830 (18)	C2—H2B	0.9411
Al1—O13	1.8853 (18)	C2—H2C	0.9411
Al1—O14	1.8740 (19)	C3—H3A	0.9302
Al1—O15	1.895 (2)	C3—H3B	0.9302
O10—H1	0.85 (4)	C3—H3C	0.9302
O10—H2	0.83 (4)		
			
O1—S1—O2	110.99 (10)	O12—Al1—O13	175.67 (9)
O1—S1—O3	110.83 (11)	O12—Al1—O14	92.21 (9)
O2—S1—O3	113.79 (10)	O12—Al1—O15	89.52 (9)
O1—S1—C1	105.26 (13)	O13—Al1—O14	91.69 (9)
O2—S1—C1	108.04 (13)	O13—Al1—O15	88.75 (9)
O3—S1—C1	107.47 (13)	O14—Al1—O15	88.20 (9)
O4—S2—O5	110.13 (11)	Al1—O10—H1	126 (2)
O4—S2—O6	112.60 (12)	Al1—O10—H2	119 (3)
O5—S2—O6	113.21 (12)	H1—O10—H2	112 (3)
O4—S2—C2	107.55 (13)	Al1—O11—H3	123 (2)
O5—S2—C2	106.32 (12)	Al1—O11—H4	121.4 (19)
O6—S2—C2	106.61 (12)	H3—O11—H4	106 (3)
O7—S3—O8	112.15 (11)	Al1—O12—H5	123 (2)
O7—S3—O9	112.23 (11)	Al1—O12—H6	115 (2)
O8—S3—O9	112.33 (11)	H5—O12—H6	119 (3)
O7—S3—C3	106.08 (13)	Al1—O13—H7	128 (3)
O8—S3—C3	107.04 (12)	Al1—O13—H8	118 (2)
O9—S3—C3	106.50 (12)	H7—O13—H8	114 (3)
O10—Al1—O11	92.23 (9)	Al1—O14—H9	127 (3)
O10—Al1—O12	89.49 (9)	Al1—O14—H10	120 (2)
O10—Al1—O13	92.26 (9)	H9—O14—H10	113 (3)
O10—Al1—O14	91.63 (9)	Al1—O15—H11	123 (3)
O10—Al1—O15	178.99 (10)	Al1—O15—H12	121 (2)
O11—Al1—O12	89.27 (8)	H11—O15—H12	107 (3)
O11—Al1—O13	86.71 (8)	S1—C1—H1A	109.5
O11—Al1—O14	175.87 (11)	H1A—C1—H1B	109.5
O11—Al1—O15	87.96 (9)		

### Hydrogen-bond geometry (Å, º)

**Table:**

*D*—H···*A*	*D*—H	H···*A*	*D*···*A*	*D*—H···*A*
O10—H1···O8^i^	0.85 (4)	1.81 (4)	2.659 (3)	174 (3)
O10—H2···O7	0.83 (4)	1.80 (4)	2.627 (3)	176 (4)
O11—H3···O1	0.84 (3)	1.78 (3)	2.615 (3)	169 (3)
O11—H4···O5^ii^	0.81 (3)	1.87 (3)	2.683 (3)	175 (3)
O12—H5···O1^iii^	0.77 (2)	1.84 (2)	2.608 (2)	171 (3)
O12—H6···O2	0.85 (3)	1.87 (3)	2.713 (2)	174 (3)
O13—H7···O3^iv^	0.79 (2)	1.87 (2)	2.648 (2)	169 (4)
O13—H8···O4^v^	0.89 (4)	1.82 (4)	2.715 (3)	177 (3)
O14—H9···O9^iii^	0.79 (4)	1.82 (4)	2.600 (3)	174 (4)
O14—H10···O4	0.88 (4)	1.84 (4)	2.714 (3)	172 (3)
O15—H11···O6^vi^	0.78 (4)	1.89 (4)	2.667 (3)	171 (4)
O15—H12···O5	0.87 (4)	1.81 (4)	2.674 (3)	169 (3)

Symmetry codes: (i) −*x*+3/2, *y*+1/2, −*z*+1/2; (ii) −*x*+1, −*y*+1, −*z*+1; (iii) *x*, *y*+1, *z*; (iv) *x*+1/2, −*y*+1/2, *z*+1/2; (v) *x*, *y*−1, *z*; (vi) −*x*+1, −*y*+2, −*z*+1.
